# Formation Control of Robotic Swarm Using Bounded Artificial Forces

**DOI:** 10.1155/2013/194280

**Published:** 2013-12-19

**Authors:** Long Qin, Yabing Zha, Quanjun Yin, Yong Peng

**Affiliations:** College of Information System and Management, National University of Defense Technology, Hunan, Changsha 410073, China

## Abstract

Formation control of multirobot systems has drawn significant attention in the recent years. This paper presents a potential field control algorithm, navigating a swarm of robots into a predefined 2D shape while avoiding intermember collisions. The algorithm applies in both stationary and moving targets formation. We define the bounded artificial forces in the form of exponential functions, so that the behavior of the swarm drove by the forces can be adjusted via selecting proper control parameters. The theoretical analysis of the swarm behavior proves the stability and convergence properties of the algorithm. We further make certain modifications upon the forces to improve the robustness of the swarm behavior in the presence of realistic implementation considerations. The considerations include obstacle avoidance, local minima, and deformation of the shape. Finally, detailed simulation results validate the efficiency of the proposed algorithm, and the direction of possible futrue work is discussed in the conclusions.

## 1. Introduction

In the past decades, multirobot systems have attracted lots of attention among researchers. With advances in communication, networking, and computing, robotic swarms are becoming more affordable in many given tasks that are too complex to be achieved by a single robot working alone. So far the possible applications range from coordinated control of UAVs [1–4] to spacecraft [5–8] and automatic vehicles [9, 10], and so forth.

The ability to achieve, maintain, and change formation is one of the fundamental prerequisites for building an effective multirobot system. Various approaches of formation control are investigated, which can be divided into three categories: the behavior-based approach, the virtual structure approach, and the potential field approach. In the behavior-based approach, a set of behaviors are assigned to each single robot and the performance of the whole group is determined by comparing the relative importance between the behaviors [11–13]. The virtual structure approach considers the entire formation as a rigid entity. Thereby the desired motion is assigned to the rigid structure and the constraint functions which relate the positions and orientations of the member robots can be defined [14, 15]. The potential field approach presents artificial forces between neighboring robots, stabilizing the system to the equilibrium manifold [16, 17]. In addition to the above classification standard, existing approaches also vary from leaderless to leader-follower and centralized to decentralized. In the leader-follower control, a real or a virtual leader is defined and its motion follows a desired trajectory. The follower robots take the leader as a reference and adjust their motion to maintain the overall formation [18, 19]. The goal of the decentralized approach is to achieve a formation while using only local information concerning positions and velocities, which is different from centralized approach relying on global information [20, 21].

Due to the physical comparability, the potential field approach which follows the law of gravitation is easier to understand. By correctly shaping potential fields, a desired behavior to a robotic swarm could be imposed [22]. Moreover, the potential field approach is generally more adaptable for building decentralized formation control algorithm [23]. Because of the intrinsic reliability of decentralized methods [22], the potential field approach is widely used in the field of swarm behavior modeling. Thus in this paper we focus on the formation control based on artificial potential forces.

Some researches focused on swarm behavior modeling [24]. Ekanayake and Pathirana [16, 25–27] proposed a scalable decentralized control algorithm to navigate a robotic swarm into a stationary shape. Their algorithm possesses remarkable robustness under external disturbances and works well in real world scenarios such as localization errors, communication range limitations, and boundedness of forces. Hengster-Movrić et al. [17] studied multiagent formation control based on bell-shaped potential function. The special feature of their potential function is its dependence on a control parameter that widens or, conversely, concentrates the effective range of interactions. This dependence is used to eliminate some potentially unwanted equilibrium states (e.g., local minima) through bifurcations [28, 29]. They also proved that the proposed controller applies in moving targets formation as well.

With a strong practicability, the above algorithms have overcame several issues which must be concerned in practical applications. However, when we attempted to apply them, we still found some unresolved problems. First, the original forces in the work of Ekanayake et al are defined in the form of upper unbounded functions. As a result, if additional rules of actuator limitations are absent, the forces will numerically overflow in certain cases. For example, when the distance between two robots is shortened significantly or one robot reaches the edge of an obstacle, the repulsion force will turn extremely large. Second, local minima of the compositive potential field still appear nearby the boundary of large obstacles. Such local minima are not taken into account by their algorithms, so the robots moving in the obstacle environment usually linger at unwanted equilibriums. Third, the formation shape itself, which is different from the robots, is considered as insensitive to the artificial forces. Thereby the shape is predefined and incapable of deforming continuously to suit the potential change.

To resolve these problems, we first define the artificial forces in the form of exponential functions which possess two types of control parameters: *magnitude factors* to determine the force magnitude and *response factors* to tune the reacting sensitivity. By carefully selecting the control parameters of these functions, the distribution of the forces can be effectively adjusted, bounding them within rational range. We further present modifications upon the control algorithm to suit realistic implementation considerations. Among these modifications we specially define the repulsion force from an obstacle which is formed by two components: one avoids the robot from collision, while the other pulls the robot to escape from the local minima nearby large obstacles. In order to make a moving shape sensitive to potential change, we build a controller upon the shape contour which provides elastic forces to compress or stretch the shape, making it autodeformable to avoid collisions. The detailed simulation results show that our control architecture can successfully resolve aforementioned problems and improve the robustness of swam behavior. However, as discussed in the conclusions, the fundamental reason which produces the local minima remained unresolved, so we highlight it as our future work and state potential solution in the end.

The outline of the paper is as follows.  Section 2 presents the swarm model and analyzes the behavior of the swarm with certain assumptions.  Section 3 discusses implementation considerations which are needed in real world scenarios.  Section 4 devises a controller acting on the shape contour so as to make the shape autodeformable, protecting it from collision with obstacles. With the simulation case studies presented in Section 5, we verify the assertions proposed in Section 2 and demonstrate the behavior of the swarm with constraints and modifications discussed in Sections 3 and 4. Finally in Section 6 we give some concluding remarks and state directions to possible future work in this area.

## 2. Swarm Model and Its Behavior Analysis

In Section 2.1 we introduce the dynamics of a swarm. In order to reduce the system complexity and highlight the basic control algorithm in the analysis phase, certain simplifications are to be considered. With the simplifications we define a series of basic artificial forces in Section 2.2 to navigate the robots. The behavior of the swarm drove by these forces is analyzed in Sections 2.3 and 2.4. The stability and convergence properties of the control algorithm are also demonstrated in the analysis.

### 2.1. The Swarm Model

We assume that (1) the robots are point masses which have no physical dimensions and follow point mass dynamics; (2) all the individuals in the same swarm should be identical in physical properties such as mass and mobility (3) all the robots are capable of instant and error free localization; and (4) within the communication network of the robots, there is no delay in any info transmission.

The state vector of a swarm of *N* robots is shown as

(1) $$\begin{array}{c} x_{i}=x_{i}^{p}⊗\left[\begin{array}{c} 1\\ 0 \end{array}\right]+x_{i}^{v}⊗\left[\begin{array}{c} 0\\ 1 \end{array}\right],\;i=1,\ldots ,N, \end{array}$$

where ⊗ denotes the Kronecker product. The entries of *x*_*i*_^*p*^, *x*_*i*_^*v*^ ∈ ℝ^2^ represent the position-like and velocity-like state variables. Thereby, the state vector of the whole swarm can be denoted as *x* = [*x*_1_,…,*x*_*N*_]^*T*^ and it is determined by

(2) $$\begin{array}{c} \dot{x}=Ax+Bu, \end{array}$$

where

(3) $$\begin{array}{c} A=I_{N}⊗\hat{A},\\ B=I_{N}⊗\hat{B}, \end{array}$$

with

(4) $$\begin{array}{c} \hat{A}=\left[\begin{array}{cc} 0 & 1\\ 0 & -\frac{b}{m} \end{array}\right],\;\hat{B}=\;\left[\begin{array}{c} 0\\ 1 \end{array}\right]. \end{array}$$

In (4), *b* is the friction coefficient which can ensure the swarm a complete stop when the forces are balanced. *m* is the mass of a robot.

### 2.2. Force Definition

The control input *u* in (2) consists of

(5) $$\begin{array}{c} u={\left[u_{1},\ldots ,u_{N}\right]}^{T}, \end{array}$$

where arbitrary entry in the vector is described as follows:

(6) $$\begin{array}{c} u_{i}=F_{i,a}+F_{i,r}+F_{i,m}=F_{i}\in {\mathbb{R}}^{2}. \end{array}$$

In our work all the components of *F*_*i*_ are built in form of exponential functions which can be generally represented as follows:

(7) $$\begin{array}{c} {F\left(k,\beta \right)=ke}^{-\beta x},\;x\in {\mathbb{R}}^{+}, \end{array}$$

where *k*, *β* ∈ ℝ^+^ are constants. Our proposed forces defined in such a form of function can be restricted in a rational range and the behavior of the swarm can be easily adjusted via selecting the parameters.

The basic artificial forces are defined as follows.

*F*_*i*,*a*_ is an attraction force on the *i*th robot from the shape which is denoted as *l*

(8) $$\begin{array}{c} F_{i,a}=k_{a}\left(1-n\left(l,x_{i}^{p}\right)\right)\int_{l}\frac{\left(z-x_{i}^{p}\right)}{||z-x_{i}^{p}||}\left(1-e^{-\beta_{a}||z-x_{i}^{p}||}\right)||dz|| \end{array}$$

*F*_*i*,*r*_ is the repulsion force on the *i*th robot from the shape *l* denoted by

(9) $$\begin{array}{c} F_{i,r}=k_{r}n\left(l,x_{i}^{p}\right)\int_{l}\frac{\left(x_{i}^{p}-z\right)}{||x_{i}^{p}-z||}e^{-\beta_{r}||z-x_{i}^{p}||}||dz||. \end{array}$$

In (8) and (9), *z* ∈ ℝ^2^ denotes a point on the shape contour. We define that *n*(*l*, *x*_*i*_^*p*^) = 1 when *x*_*i*_^*p*^ is inside the shape, and *n*(*l*, *x*_*i*_^*p*^) = 0 when it is outside. The mutual exclusion ensures that *F*_*i*,*a*_ will vanish only if the robot is inside *l*, and *F*_*i*,*r*_ will vanish only if the robot is outside *l*.

*F*_*i*,*m*_ in (10) refers to the resultant repulsion force acting on the *i*th robot from the remaining swarm robots. That is,

(10) $$\begin{array}{c} F_{i,m}=k_{m}\overset{N}{\underset{j=1,j\neq i}{\sum }}\frac{\left(x_{i}^{p}-x_{j}^{p}\right)}{||x_{i}^{p}-x_{j}^{p}||}e^{-\beta_{m}||x_{i}^{p}-x_{j}^{p}||}. \end{array}$$

From (8) to (10), *β*_*a*_, *β*_*r*_, and *β*_*m*_ adjust reacting sensitivity, *k*_*a*_,  *k*_*r*_, and *k*_*m*_ determine the magnitude of the force, (*z* − *x*_*i*_^*p*^)/||*z* − *x*_*i*_^*p*^||,  (*x*_*i*_^*p*^ − *z*)/||*x*_*i*_^*p*^ − *z*||,  (*x*_*i*_^*p*^ − *x*_*j*_^*p*^)/||*x*_*i*_^*p*^ − *x*_*j*_^*p*^|| ∈ ℝ^2^ are unit vectors determining the force directions.

Generally speaking, *F*_*i*,*a*_ and *F*_*i*,*r*_ are the forces navigating the robots towards the desired shape and evenly spreading them inside the shape. *F*_*i*,*m*_ avoids the intermember collisions.

### 2.3. Analysis of the Swarm Behavior

As *F*_*i*,*a*_ and *F*_*i*,*r*_ are mutually exclusive and do not work simultaneously, the swarm behavior and the stability of the controller can be analyzed in each instance separately. Definitions defined in (11) will be employed by the analysis.

Consider

(11) $$\begin{array}{c} z_{cm}=\frac{\sum_{i=1}^{N}x_{i}^{p}}{N},\;\;z_{c}=\frac{\int_{l}z||dz||}{c_{l}},\;\;c_{l}=\int_{l}||dz|| \end{array}$$

*z*_*cm*_, *z*_*c*_, and *c*_*l*_ represent the center of mass of the swarm, the center of mass of the shape contour, and length of the shape contour.

#### 2.3.1. Motion of the Swarm and Its Members outside the Formation Shape

When all the robots are operating outside the target shape, the swarm can be viewed as one object affected by resultant force described as follows:

(12) $$\begin{array}{c} F=F_{a}+F_{m}+F_{f}. \end{array}$$

*F*_*m*_ is the resultant intermember force and equals to zero; that is,

(13) $$\begin{array}{c} F_{m}=\overset{N}{\underset{i=1}{\sum }}k_{m}\;\overset{N}{\underset{j=1,j\neq i}{\sum }}\frac{\left(x_{i}^{p}-x_{j}^{p}\right)}{||x_{i}^{p}-x_{j}^{p}||}e^{-\beta_{m}||x_{i}^{p}-x_{j}^{p}||}=0. \end{array}$$

*F*_*a*_ is the attraction force from the target shape

(14) $$\begin{array}{c} F_{a}=k_{a}\overset{N}{\underset{i=1}{\sum }}\;\int_{l}\frac{\left(z-x_{i}^{p}\right)}{||z-x_{i}^{p}||}\left(1-e^{-\beta_{\alpha }||z-x_{i}^{p}||}\right)||dz||. \end{array}$$

With definitions in (11), we can infer from (14) that:

(15) Fa<ka∑i=1N ∫l(z−xip)||z−xip||(1−(1−βα||z−xip||))||dz||=ka∑i=1N ∫lβα(z−xip)||dz||=βαclNka(zc−zcm).

From (4), we know that the friction force acting on the *i*th robot is *F*_*i*,*f*_ = − (*b*/*m*)*x*_*i*_^*v*^, so the total friction force acting on the swarm can be calculated as follows:

(16) $$\begin{array}{c} F_{f}=-\left(\frac{Nb}{m}\right)\left({\dot{z}}_{cm}\right). \end{array}$$

We define *ε* = (*z*_*cm*_ − *z*_*c*_) with $\dot{\varepsilon }={\dot{z}}_{cm}$ and $\ddot{\varepsilon }={\ddot{z}}_{cm}$. According to (12), the dynamics of the swarm possess a property as follows:

(17) Nmε¨<−Nb(z˙cm)+βαclNka(zc−zcm)⇒ε¨+(bm)ε˙+βαclkaε<0.

With (17) we can conclude Theorem 1 as follows.

**Theorem 1**

Consider the swarm model whose dynamics of motion has property described as (17), the motion of the center of mass of the swarm (*z*_*cm*_) is toward the center of mass of the shape contour (*z*_*c*_).

**Proof**

Taken Lyapunov function $V_{\varepsilon }=\left(1/2\right)\left(m\dot{\varepsilon }{\dot{\varepsilon }}^{T}+{\alpha c}_{l}k_{a}\varepsilon \varepsilon^{T}\right)$, its differential coefficient on time *t*. That is, $\dot{V_{\varepsilon }}$ can be deducted as follows:

(18) V˙ε=mε˙ε¨T+βαclkaεε˙T<−(bm)ε˙ε˙T−βαclkaεε˙T+βαclkaεε˙T⇒V˙ε<−(bm)||ε˙||2.

So ${\dot{V}}_{\varepsilon }<0$, for any $||\dot{\varepsilon }||\neq 0$, the only invariant point is ($\varepsilon =\dot{\varepsilon }=0$), by using Lyapunov's method extended by Lãsalle and Lefschetz [30], the Theorem 1 is proved to be true.

Intuitively, Theorem 1 argues that when all the robots are outside the shape, the center of mass of the swarm will move towards the center of mass of the target shape.

The characteristic polynomial of system (17) is

(19) $$\begin{array}{c} \lambda^{2}+\frac{b}{m}\lambda +\beta_{\alpha }c_{l}k_{a}=0, \end{array}$$

and it is clear that, in order to archive damped motion which ensures the swarm is a smooth movement, the conditions *b* > 0, *k*_*a*_ > 0 and $b\geq 2\sqrt{m{\beta_{\alpha }c}_{l}k_{a}}$ must be satisfied.

Theorem 1 only states the dynamics of the swarm as one object, while the behavior of every single robot remains uncertain. Next the behavior of an individual robot will be investigated.

For the *i*th robot, its dynamics of motion can be described as follows:

(20) $$\begin{array}{c} {m\ddot{x}}_{i}^{p}=F_{i,a}+F_{i,m}+F_{i,f}. \end{array}$$

To the *F*_*i*,*a*_ defined in (8), where (1 − *n*(*l*, *x*_*i*_^*p*^)) = 1, we have

(21) Fi,a<ka∫l(z−xip)||z−xip||(1−(1−βα||z−xip||))||dz||=βαka∫l(z−xip)||dz||⇒Fi,a<βαkacl(zc−xip).

For *F*_*i*,*m*_ we define *d*_*i*,*j*_ = ||*x*_*i*_^*p*^ − *x*_*j*_^*p*^||, with (11) and the friction force definition, the dynamics of motion of *i*th robot is described as

(22) mx¨ip<βαkacl(zc−xip)+km∑j=1,j≠iN(xip−xjp)||xip−xjp||e−βγ||xip−xjp||−bx˙ip.

With definition *v*_*i*_ = (*x*_*i*_^*p*^ − *z*_*cm*_), (22) can be rewritten as

(23) m(x¨ip−z¨cm)+m(z¨cm−z¨c) <−βαkacl(xip−zcm)−βαkacl(zcm−zc)  −b(x˙ip−z˙cm)−b(z˙cm−z˙c)  +km∑j=1,j≠iN(xip−xjp)||xip−xjp||e−βγ||xip−xjp||  ⇒mv¨i+bv˙i+βαkaclvi−Fi,m<0.

By definition of *δ* = min⁡⁡{*d*_*i*,*j*_ *i*, *j* ∈ *N*  and  *i* ≠ *j*}, we have

(24) $$\begin{array}{c} ||F_{i,m}||<k_{m}||\overset{N}{\underset{j=1,j\neq i}{\sum }}\frac{\left(x_{i}^{p}-x_{j}^{p}\right)}{\delta }||=\;\frac{k_{m}\left(N-1\right)}{\delta }||x_{i}^{p}-z_{cm}^{i}||, \end{array}$$

where

(25) $$\begin{array}{c} z_{cm}^{i}=\frac{\sum_{j=1,j\neq i}^{N}x_{j}^{p}}{N-1}. \end{array}$$

Given the definition of *z*_*cm*_ and *z*_*cm*_^*i*^,

(26) xip−zcm=∑j=1,j≠iN(xip−  xjp)N,xip−zcmi=∑j=1,j≠iN(xip−  xjp)N−1⇒||xip−zcmi||||xip−zcm||=1−1N<1,

So from (24) we state that

(27) $$\begin{array}{c} ||F_{i,m}||<\frac{k_{m}\left(N-1\right)}{\delta }||x_{i}^{p}-z_{cm}||. \end{array}$$

In order to form the member robots into a coherent swarm, the artificial forces in right hand side of (20) must drive each robot toward the center of mass of the swarm, and this is just what Theorem 2 concludes as follows.

**Theorem 2**

Consider a robot *i* staying outside the shape, if *k*_*a*_/*k*_*m*_ > (*N* − 1)/*δc*_*l*_ is satisfied, then its motion is toward the center of the swarm *z*_*cm*_.

**Proof**

Choosing a Lyapunov function as

(28) $$\begin{array}{c} V_{i}=\frac{1}{2}m{\dot{v}}_{i}{{\dot{v}}_{i}}^{T}+\frac{1}{2}v_{i}v_{i}^{T}\left({\beta_{\alpha }k}_{a}c_{l}-\frac{k_{m}\left(N-1\right)}{\delta }\right)>0, \end{array}$$

through taking derivatives, ${\dot{V}}_{i}$ is bounded by

(29) $$\begin{array}{c} {\dot{V}}_{i}\leq -\frac{b}{m}{||{\dot{v}}_{i}||}^{2}+\left(||F_{i,m}||-\frac{k_{m}\left(N-1\right)}{\delta }||v_{i}||\right)||{\dot{v}}_{i}||, \end{array}$$

and it is clear that with (27), the second item of the right hand side of (29) is negative, so we have

(30) $$\begin{array}{c} {\dot{V}}_{i}\leq -\frac{b}{m}{||{\dot{v}}_{i}||}^{2}, \end{array}$$

which proves the theorem.

Here with Theorems 1 and 2 we argue that with the artificial forces defined in Section 2.2, the swarm staying outside the contour can move toward the target shape and at the same time cohere the robots within certain interdistance.

#### 2.3.2. Motion of the Swarm and Its Members inside the Formation Shape

In this section we proof that when a robot is inside a shape contour which is symmetrical over two or more straight lines and if it is controlled by *F*_*i*,*r*_ defined in (9), then the intersection point of those lines will be the equilibrium point where the robot will stay still after a certain time span.

The lemma we used is given without proof. Readers who are interested in this can find more detail in [16].  Lemma 3 use *odd* and *even* function properties to derive a property of a complex function which is symmetrical over real axis of the complex plane, as described below.

**Lemma 3**

For a closed contour *l*(*θ*) and functions *f*_1_(*θ*) ∈ ℝ^+^, *f*_2_(*θ*) ∈ *C* for *θ* ∈ [0,2*π*], with the following properties:

(31) $$\begin{array}{c} l\left(\theta \right)=l^{*}\left(2\pi -\theta \right),\;\;f_{1}\left(\theta \right)=f_{1}\left(2\pi -\theta \right),\\ f_{2}\left(\theta \right)=l\left(\theta \right)-C_{R},\;C_{R}\in \mathbb{R}. \end{array}$$

We define *ℑ*(·) the imaginary component of a complex number; then the following statement holds:

(32) $$\begin{array}{c} ℑ\left(\overset{2\pi }{\underset{0}{\int }}f_{1}\left(\theta \right)f_{2}\left(\theta \right)d\theta \right)=0. \end{array}$$

As shown in (9), *F*_*i*,*r*_ = *k*_*r*_∫_*l*_((*x*_*i*_^*p*^ − *z*)/||*x*_*i*_^*p*^−*z*||)*e* ^−*β*_*r*_||*z*−*x*_*i*_^*p*^||^||*dz*|| consists of two major components, (*x*_*i*_^*p*^ − *z*)/||*x*_*i*_^*p*^ − *z*|| defines the direction of the force, and *e* ^−*β*_*r*_||*z*−*x*_*i*_^*p*^||^ determines the magnitude of the force. The expression for *F*_*i*,*r*_ is the contour integral of

(33) $$\begin{array}{c} k_{r}\overset{K}{\underset{k=1}{\sum }}\;\frac{\left(x_{i}^{p}-z_{k}\right)}{||x_{i}^{p}-z_{k}||}e^{-\beta_{r}||z_{k}-x_{i}^{p}||},\;\text{where}\;z_{k}\in l, \end{array}$$

for

(34) $$\begin{array}{c} \forall k,\;\text{when}\;k\to \infty ,\;z_{k}-z_{k-1}=z_{k+1}-z_{k}. \end{array}$$

We define *f*(*z*, *x*_*i*_^*p*^) = (1/||*x*_*i*_^*p*^−*z*||)*e* ^−*β*_*r*_||*z*−*x*_*i*_^*p*^||^. Using polar coordinate representation for complex plane, *z*_*k*_ = *l*(*θ*) and *F*_*i*,*r*_ can be rewritten as

(35) $$\begin{array}{c} F_{i,r}=k_{r}\overset{2\pi }{\underset{0}{\int }}f\left(l\left(\theta \right),x_{i}^{p}\right)\left(x_{i}^{p}-l\left(\theta \right)\right)||\dot{l}\left(\theta \right)||d\theta . \end{array}$$

If the contour *l*(*θ*) with *θ* ∈ [0,2*π*) is symmetrical over the real axis and the *i*th robot is staying along on the real axis (i.e., *l*(*θ*) = l*(2*π* − *θ*) and *ℑ*(*x*_*i*_^*p*^) = 0); then we have

(36) f1(θ)=f(l(θ),xip)||l˙(θ)||=f(l(2π−θ),xip)||l˙(2π−θ)||,f2(θ)=(xip−l(θ)).

Thus using Lemma 3, *ℑ*(*F*_*i*,*r*_) = 0 is proved, which is used to prove Theorem 4 as described below.

**Theorem 4**

Given a robot inside a shape contour *l*(*θ*) with the following properties:

1. *l*(*θ*) = l*(2*π* − *θ*),
2. *ℑ*(*x*_*i*_^*p*^) = 0.

Then *ℑ*(*F*_*i*,*r*_) = 0.

The above assertion can also be extended to determine the behavior of the whole swarm when all the robots are inside the shape, through viewing the swarm as one object, the center of mass of the swarm will finally move to the equilibrium point, and the whole swarm will evenly be distributed inside the contour, as described in Theorem 5.

**Theorem 5**

Given a shape contour *l*(*θ*) with two or more symmetric axes. Then for a robot i or the center of mass of a swarm located at the intersection of those axes,  *F*_*i*,*r*_ = 0.

**Proof**

Consider *l*(*θ*) has *N* symmetric axes (*k*_1_,…, *k*_*N*_) forming *σ*_*i*_ ∈ [0,2*π*) angles to the real axis, from Theorem 4 we state that when a member or the center of mass of a swarm is located on the intersection of those axes, the repulsion force *F*_*i*,*r*_ is in the form of

(37) $$\begin{array}{c} F_{i,r}=\mu_{1}e^{i\sigma_{1}}=\cdots =\mu_{N}e^{i\sigma_{N}}, \end{array}$$

where *μ*_1_,…, *μ*_*N*_ ∈ ℝ, because *σ*_1_ ≠ *σ*_2_,…, ≠*σ*_*N*_; this implies *μ*_1_ = … = *μ*_*N*_ = 0, which means that *F*_*i*,*r*_ = 0.

### 2.4. Discussion on Transitional State

In this section we focus on the transitional state in which a part of the swarm is inside the contour, while the rest of them stay outside. From Theorem 2, when *δ* increase,the condition *k*_*a*_/*k*_*m*_ > (*N* − 1)/*δc*_*l*_ will be satisfied and the attraction force *F*_*i*,*a*_ acting on members who stay outside the contour will be dominating. Thus, the remaining part can still move toward the target shape. However, when a robot is staying closer but outside the contour and the contour is not large enough to accommodate all the robots; then *F*_*i*,*m*_ turns larger than *F*_*i*,*a*_, which will lead the robot in an unwanted equilibrium state. This problem can be eliminated by carefully tuning *k*_*a*_ and *k*_*m*_ to suit the swarm size or, as described in the next section, to modify *F*_*i*,*a*_ to get uniform distribute of the forces near by the contour.

## 3. Implementation Considerations

When implementing the proposed control algorithm in a real world scenario, the simplifications assumed in Section 2 will no longer hold. So in Section 3.1 we discuss certain modifications upon the proposed basic forces, which ensure the robustness of the swarm behavior in the presence of realistic implementation considerations. In Section 3.2 we investigate the rules of selecting proper control parameters so as to suit certain applications. Moreover, we show in Section 3.3 that by certain coordinate transformations the controller can be applied to moving targets formation as well.

### 3.1. Controller Modification for Practical Implementation

#### 3.1.1. Physical Dimensions of Robots

**Figure 1:**
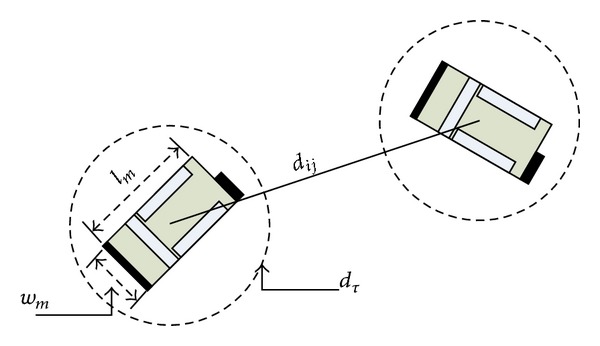
Car-like robots with turning diameter.

Here the robots are no longer considered as point masses. For general case, a robot with physical dimensions possesses length (*l*_*m*_) and width (*w*_*m*_), where *l*_*m*_ > *w*_*m*_ (see Figure 1).

The operational diameter of the robot is then defined as *d*_*t*_ = *τ*_*t*_ × *l*_*m*_, where *τ*_*t*_ > 1. *τ*_*t*_ is the safe factor for collision avoidance. By selecting *τ*_*t*_ the distribution of the repulsion force can be adjusted to suit the maximum speed of the motion. Thereby the modified intermember repulsion force can be stated as follows:

(38) $$\begin{array}{c} {\tilde{F}}_{i,m}=k_{m}\overset{N}{\underset{j=1,j\neq i}{\sum }}\frac{\left(x_{i}^{p}-x_{j}^{p}\right)}{||x_{i}^{p}-x_{j}^{p}||}e^{-\beta_{m}(||x_{i}^{p}-x_{j}^{p}||-{\;d}_{t})}. \end{array}$$

**Figure 2:**
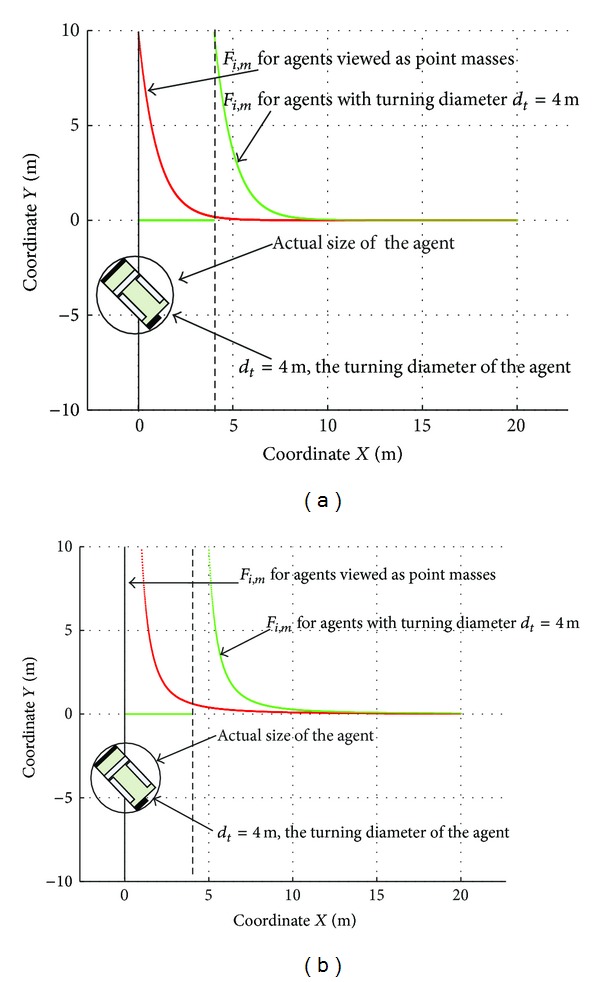
Comparison of ${\tilde{F}}_{i,m}$ and *F*_*i*,*m*_.

The comparison between ${\tilde{F}}_{i,m}$ and *F*_*i*,*m*_ is shown in Figure 2(a). Additionally we give ${\tilde{F}}_{i,m}$ and *F*_*i*,*m*_ defined in [24] as counterpart in Figure 2(b). It is shown that the magnitude of ${\tilde{F}}_{i,m}$ and *F*_*i*,*m*_ defined in our work is bounded, which can avoid numerical overflow by selecting proper control parameters.

#### 3.1.2. Obstacle Avoidance

Obstacle avoidance is one of the most important aspects in practical implementation of the algorithm [31–37]. In general the repulsion forces from obstacles only effect within a finite distance; that is, the sensing radius *R*_*s*_ of a member and its magnitude should be limited too. So repulsion force acting on *i*th member from obstacle *O* is presented as follows:

(39) $$\begin{array}{c} F_{i,o}=k_{o}\int_{l_{o}}f_{i,o}\left(z\right)||dz||, \end{array}$$

(40) $$\begin{array}{c} f_{i,o}=\{\begin{array}{cc} \frac{\left(x_{i}^{p}-z\right)}{||x_{i}^{p}-z||}e^{-\beta_{o}||z-x_{i}^{p}||}, & \text{when}\;\left(||z-x_{i}^{p}||\right)\in \left(0,R_{s}\right],\\ 0, & \text{when}\;\left(||z-x_{i}^{p}||\right)>R_{s}, \end{array} \end{array}$$

where *k*_*o*_ denotes the weight determining the magnitude of the force. *l*_*o*_ is the obstacle's simply-closed contour and *z* ∈ ℝ^2^ is an arbitrary point on that contour.

**Figure 3:**
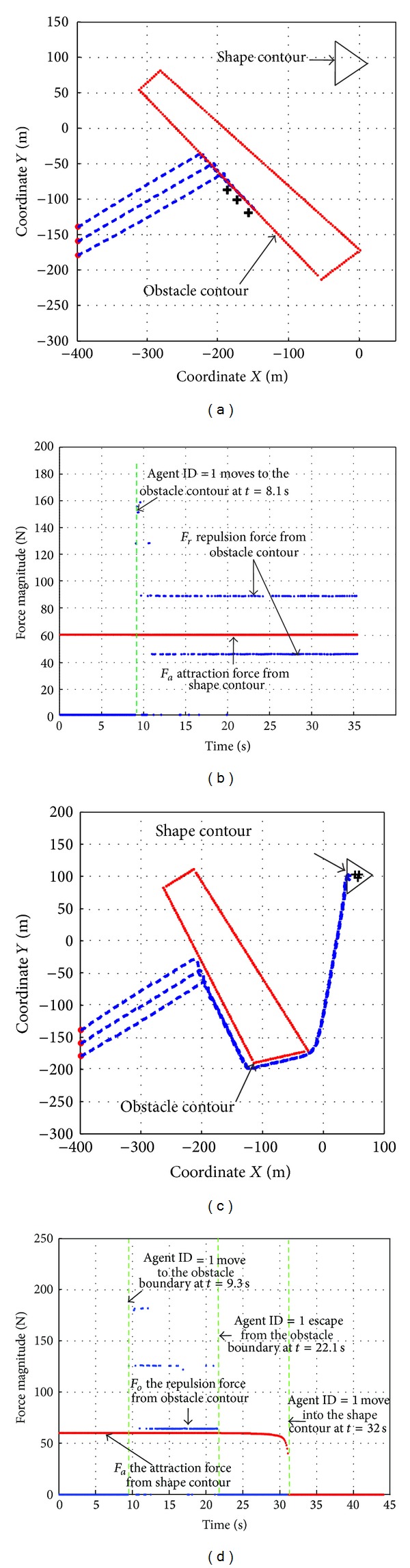
Comparison on original and modified *F*_*i*,*o*_ on impasse resolution.

However, only using (40) to determine the repulsion force may lead to local minima near by the boundary of an obstacle, because *F*_*i*,*o*_ and *F*_*i*,*a*_ may get balanced.  Figure 3(a) shows that three robots lingered near by the obstacle boundary. The reason is that, as Figure 3(b) shows, when the robots move to the edge of the obstacle, *F*_*i*,*a*_ (represented by red line) together with *F*_*i*,*o*_ (represented by blue dot line) drive the robot into an unwanted impasse, where *F*_*i*,*a*_ was counteracted by *F*_*i*,*o*_, so there will be no actuator to navigate the robot heading for the destination.

To resolve this problem we decompose *f*_*i*,*o*_ into two components, pointing at directions (*x*_*i*_^*p*^ − *z*)/||*x*_*i*_^*p*^ − *z*|| and $\left[\begin{array}{cc} 0 & 1\\ -1 & 0 \end{array}\right]\left(\left(x_{i}^{p}-z\right)/||x_{i}^{p}-z||\right)$, respectively, so *f*_*i*,*o*_ is rewritten as follows (where *I*_2×2_ denotes identity matrix):

(41) $$\begin{array}{c} f_{i,o}=\{\begin{array}{cc} \frac{1}{2}\left(I_{2\times 2}+\left[\begin{array}{cc} 0 & 1\\ -1 & 0 \end{array}\right]\right)\frac{\left(x_{i}^{p}-z\right)}{||x_{i}^{p}-z||}e^{-\beta_{o}||z-x_{i}^{p}||}, & \\ \text{when}\;\left(||z-x_{i}^{p}||\right)\in \left(0,R_{s}\right], & \\ 0,\text{when}\;\left(||z-x_{i}^{p}||\right)>\;R_{s}. & \end{array} \end{array}$$

The part multiplied by $\left[\begin{array}{cc} 0 & 1\\ -1 & 0 \end{array}\right]\left(\left(x_{i}^{p}-z\right)/||x_{i}^{p}-z||\right)$ in (41) generates a force pulling robot to move along the boundary and escape them from the impasse. Figures 3(c) and 3(d) are counterparts of the same scene but using (41) to calculate *F*_*i*,*o*_. These two figures show that the whole swarm can successfully escape the local minima and get into the shape contour.

#### 3.1.3. Actuator Limitations

A robot has its limitation on acceleration and velocity, so the force definitions must be modified to eliminate unrealistic accelerations. Considering the mass of the *i*th member is *m*, and its upper threshold of acceleration is *a*_*m*_; then the controller is shown as

(42) $$\begin{array}{c} u_{i}={\tilde{F}}_{i}^{R}, \end{array}$$

where

(43) $$\begin{array}{c} {\tilde{F}}_{i}^{R}=\{\begin{array}{cc} F_{i}, & \text{if}\;F_{i}<m\times a_{m},\\ m\times a_{m}, & \text{if}\;F_{i}\geq m\times a_{m}. \end{array}\; \end{array}$$

We use velocity limiting function introduced in [24] as,

(44) $$\begin{array}{c} ||{\tilde{x}}_{i}^{v}||=\;\{\begin{array}{cc} ||x_{i}^{v}||, & \text{if}\;||x_{i}^{v}||<v_{\max},\\ v_{\max}, & \text{if}\;||x_{i}^{v}||<v_{\max}, \end{array} \end{array}$$

the term *v*_max⁡_ represents maximum velocity that the *i*th member can achieve. $||{\tilde{x}}_{i}^{v}||$ is the desired speed.

#### 3.1.4. Chattering Effect

**Figure 4:**
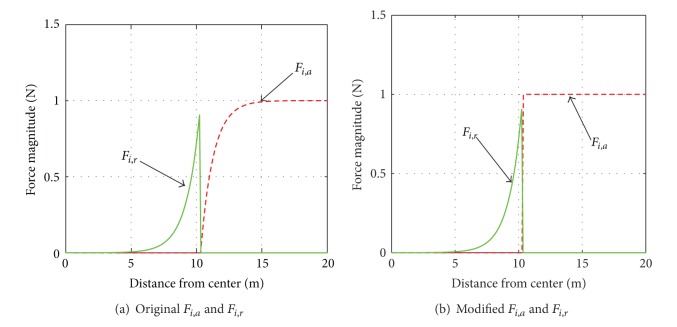
Distribution of original and modified *F*_*i*,*a*_ and *F*_*i*,*r*_ nearby the shape contour.

A drawback of the basic controller is the discontinuous nature of the basic artificial forces (i.e., *F*_*i*,*a*_ and *F*_*i*,*r*_ defined in Section 2). As shown in Figure 4(a), the forces have a significant variation at the boundary. If the shape area is relatively small, the repulsion force from other robots which are already inside the contour will prevent the others from passing through the edge. To tackle this problem, the force defined in (8) is modified to add a term which can compensate *F*_*i*,*a*_ when the robot is getting closer to the shape contour. The modified *F*_*i*,*a*_ is as follows:

(45) Fi,a=(1−n(l,xip))∫l(z−xip)||z−xip||×[ka(1−e−βa||z−xip||)  +  kace−βac||xip−z||]||dz||.

In (45), *k*_*ac*_ determine the magnitude of the additional force. By tuning parameter *β*_*ac*_, the operating range of this term can be limited in a short distance from the contour.  Figure 4 shows that compared with original *F*_*i*,*a*_ (Figure 4(a)), modified *F*_*i*,*a*_ (Figure 4(b)) has preferable uniformity in either side of the shape contour. For the sake of simplicity, the values of the parameters are set as *k*_*a*_ = 1,  *k*_*r*_ = 1,  *β*_*a*_ = 1,  *β*_*ac*_ = 1, and *β*_*r*_ = 1.

### 3.2. Adaptation of Control Parameters

The behavior of the forces defined in this paper is mainly determined by the value of the control parameters which can be classified into two types: *magnitude factors *(*k*_*a*_, *k*_*ac*_, *k*_*r*_, *k*_*m*_, and *k*_*o*_) and *response factors* (*β*_*a*_, *β*_*ac*_, *β*_*r*_, *β*_*m*_, and *β*_*o*_). The basic rules of determining values of these parameters are summarized in this subsection.

#### 3.2.1. Magnitude Factors

For the determination on magnitude factors *k*_*a*_, *k*_*ac*_, *k*_*r*_, and *k*_*o*_, an uniform representation is defined as follows:

(46) $$\begin{array}{c} k_{*}=\frac{F_{\text{upper}}}{\int_{l}||dz||}=\frac{F_{\text{upper}}}{c_{l}}, \end{array}$$

where *F*_upper_ ∈ ℝ^+^ is the total force that a shape or obstacle can generate. From the definitions of *F*_*i*,*a*_, *F*_*i*,*r*_, and *F*_*o*_ it is obvious that *F*_∗_ ≤ *k*_∗_, where the symbol ∗ can be replaced by *a*, *r*, and *o*.

For *k*_*m*_ we state that

(47) $$\begin{array}{c} k_{m}=N\times F_{\text{per}}, \end{array}$$

where *F*_per_ ∈ ℝ^+^ is the unit repulsion force and *N* is the count of member in the swarm.

With (46) and (47), the proposed forces can be limited by certain upper bound.

#### 3.2.2. Response Factors

The definitions of the artificial forces show that the response factors *β*_∗_ can adjust the force's reacting sensitivity. To set the value we have

(48) $$\begin{array}{c} \beta_{*}=\frac{1}{R_{s}}, \end{array}$$

where the symbol ∗ can be replaced by *a*, *r*,*m*, *o*, and *R*_*s*_ ∈ ℝ^+^ to represent the constant of the robot's sensing radius.

### 3.3. Moving Targets Formation

When a target shape is executing maneuver, there is a frame of reference *x*′*o*′*y*′ tightly connecting with it. The direction of the swarm's velocity coincides with the *y*-axis and points on the shape contour are therefore fixed in such a frame. At time *t*, the position-like state vector of the *i*th member under the stationary (global) frame of reference *xoy* is *x*_*i*_^*p*^(*t*) = [*x*, *y*], by adopting displacement and rotation transformation as shown in (49), ${\tilde{x}}_{i}^{p}\left(t\right)$ under *x*′*o*′*y*′ can be calculated by

(49) $$\begin{array}{c} {\tilde{x}}_{i}^{p}\left(t\right)=\left[\begin{array}{c} x\cos\theta +y\sin\theta +\Delta x\\ -x\sin\theta +y\cos\theta +\Delta y \end{array}\right], \end{array}$$

where *θ* denotes the angle of rotation of *x*′*o*′*y*′ on *xoy* and [Δ*x*, Δ*y*]^*T*^ is the displacement vector pointing from origin *o* to *o*′.

Consider that the mode of a 2D vector is invariant under coordinate transformations, *F*_*i*,*a*_ and *F*_*i*,*r*_ under frame *x*′*o*′*y*′ can be calculated via replacing *x*_*i*_^*p*^, *z* in (8) and (9) with ${\tilde{x}}_{i}^{p}\left(t\right),\;\;\tilde{z}$.

**Proof**

Since

(50) [x~ip(t)−z~]T=[(x−zx)cos⁡⁡θ+(y−zy)sinθ−(x−zx)sin⁡θ+(y−zy)cos⁡θ]⇒||[x~ip(t)−z~]T||=||[xip(t)−z]T||.

So the scalar quantity of *F*_*i*,*a*_ and *F*_*i*,*r*_ is invariant under coordinates transformation defined by (49).

Here the forces generated from a moving formation was denoted as ${\overset{-}{F}}_{i,a}$ and ${\overset{-}{F}}_{i,r}$ are rewritten as

(51) F−i,a=ka[cos⁡θ00−sinθ]×[∫lx~ip(t)−z~||x~ip(t)−z~||(1−e−βa||x~ip(t)−z~||)||dz~||],

(52) F−i,r=kr[cos⁡θ00−sinθ]∫lx~ip(t)−z~||x~ip(t)−z~||e−βr||z~−x~ip(t)||||dz~||.

By replacing original force definitions presented in (8) and (9) with (51) and (52) respectively, we can analogously analyze the swarm behavior and prove that the relative assertions proposed in Section 2 also hold for ${\overset{-}{F}}_{i,a}$ and ${\overset{-}{F}}_{i,r}$.

## 4. Analysis of the Controller Acting on the Shape Contour

Most of the formation control strategies that are based on artificial potential field only control the overall geometry, while recently some novel strategies have been put forward to control the exact shape of the formation [38–40].

When a moving target shape is about to pass through a narrow channel or collide with an obstacle, it is expected that the shape can be autodeformable to suit potential field change and therefore prevent the robots within it from collisions with obstacles. Moreover, in order to accommodate enough space to the whole swarm, it must be ensured that the area of the shape will not change during the deformation phase.

**Figure 5:**
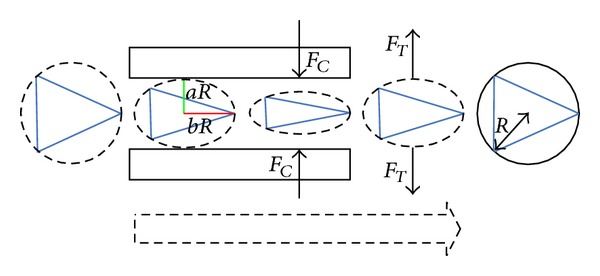
The deforming procedure of the shape contour in the case of passing through a narrow channel.

To achieve this purpose, in this section we present a controller on the shape contour which is sensitive to the potential change. The controller generates two forces acting on the shape contour: the pressure *F*_*C*_ from obstacles and the tension *F*_*T*_ generated by the contour itself. We analyze the behavior of this controller via a case in which a robotic swarm is passing through a narrow channel, as shown in Figure 5.

We define *R* as radius of the smallest enclosing circle of the shape. When the circle is compressed into an ellipse; that is,

(53) $$\begin{array}{c} \frac{X^{2}}{a^{2}}+\frac{Y^{2}}{b^{2}}=R^{2}, \end{array}$$

where we have

(54) $$\begin{array}{c} \left[\begin{array}{c} X\\ Y \end{array}\right]=\left[\begin{array}{cc} a & 0\\ 0 & b \end{array}\right]\times \left[\begin{array}{c} x\\ y \end{array}\right], \end{array}$$

the area of the resulting ellipse is *S* = *π* × *abR* ^2^. It is clear that if *ab* = 1, the area of the circle will be invariant under the compression.  Theorem 6 states that the shape within the circle also keeps its area invariant during the compression.

**Theorem 6**

Given a shape presented as a convex polygon *P* with *N* ≥ 3 vertex, if a compression satisfies condition *ab* = 1 on its smallest enclosing circle, then the area of *P* is invariant.

**Proof**

*P* can be divided into *N*-2 triangles, for each one of them (denotes as Δ) with vertexes {*v*_1_, *v*_2_, *v*_3_},

(55) $$\begin{array}{c} S_{\Delta c}=\frac{1}{2}||\begin{array}{ccc} X_{v1} & Y_{v2} & 1\\ X_{v2} & Y_{v3} & 1\\ X_{v3} & Y_{v3} & 1 \end{array}||=\frac{1}{2}||\begin{array}{ccc} ax_{v1} & by_{v2} & 1\\ ax_{v2} & by_{v3} & 1\\ ax_{v3} & {by}_{v3} & 1 \end{array}||=abS_{\Delta }, \end{array}$$

where  *S*_Δ*c*_ and  *S*_Δ_ are the area after and before compression, respectively. If *ab* = 1, then *S*_Δ*c*_ = *S*_Δ_. So

(56) $$\begin{array}{c} S_{pc}=\overset{N-2}{\underset{i=1}{\sum }}S_{\Delta c}^{i}=ab\overset{N-2}{\underset{i=1}{\sum }}S_{\Delta }^{i}=abS_{p}=S_{p}, \end{array}$$

which supports the assertion.

During the compression phase, the smallest enclosing circle itself will generate a tensile force *F*_*T*_ which tends to pull the shape contour back to its original state, which is represented as

(57) $$\begin{array}{c} F_{T}=\lambda_{c}\Delta x, \end{array}$$

where Δ*x* denotes the compression ratio, and *λ*_*c*_ ∈ ℝ^+^ the range.

We define the width of the channel is *w*_*c*_, the diameter of the smallest enclosing circle is *d*; then the compression force can be calculated by (58) as

(58) $$\begin{array}{c} F_{C}=\{\begin{array}{cc} \lambda_{c}\times \frac{\left(d-w_{c}\right)}{d}, & \text{if}\;\;w_{c}<d,\\ 0, & \text{if}\;\;w_{c}\geq d. \end{array} \end{array}$$

Consider state vector

(59) $$\begin{array}{c} \left[\begin{array}{c} x_{1}\\ x_{2} \end{array}\right]=\left[\begin{array}{c} 1-a\\ {\dot{x}}_{1} \end{array}\right], \end{array}$$

then the dynamics of motion of [*x*_1_, *x*_2_]^*T*^ will be

(60) $$\begin{array}{c} \left[\begin{array}{c} {\dot{x}}_{1}\\ {\dot{x}}_{2} \end{array}\right]=\;\left[\begin{array}{cc} 0 & 1\\ -\lambda_{c} & -b_{c} \end{array}\right]\left[\begin{array}{c} x_{1}\\ x_{2} \end{array}\right]+\;\left[\begin{array}{c} 0\\ 1 \end{array}\right]F_{C}, \end{array}$$

where $b_{c}=\sqrt{4\lambda_{c}+1}$ is the friction factor.

From (58) and (60), we can get the transfer function of a second-order system as follows:

(61) $$\begin{array}{c} T\left(s\right)=\frac{\lambda_{c}\left(\left(d-w_{c}\right)/d\right)}{s^{2}+b_{c}s+\lambda_{c}}=\frac{\lambda_{c}\left(\left(d-w_{c}\right)/d\right)}{s^{2}+s\sqrt{4\lambda_{c}+1}+\lambda_{c}}, \end{array}$$

its damping ratio is $\zeta =\;\left(1/2\right)\sqrt{4+\left(1/\lambda_{c}\right)}>1$, so it is an over damping system with adjusting time $t_{s}=8/\sqrt{4\lambda_{c}+1}$, and the compression matrix is

(62) $$\begin{array}{c} \left[\begin{array}{cc} a & 0\\ 0 & b \end{array}\right]=\left[\begin{array}{cc} 1-x_{1} & 0\\ 0 & \frac{1}{1-x_{1}} \end{array}\right]. \end{array}$$

So an arbitrary point *z* on the shape contour will be deformed into a new position as $\tilde{z}=\left[\begin{array}{cc} a & 0\\ 0 & b \end{array}\right]\times z$ and the attraction and repulsion forces generated by the shape can be recomputed and updated to drive the robots.

## 5. Simulation Results and Discussion

In this section we first test the proposed control algorithm and the swarm behavior by three basic cases: formation convergence (in Section 5.1), formation transition (in Section 5.2), and obstacle avoidance (in Section 5.3). Within these cases the basic swarm model proposed in Section 2 and the modified one investigated in Section 3 are both taken into account, which by contrast highlights the improvement of the latter when it is working under certain restrictions in the real world scenario.

In Section 5.4 we apply the control algorithm to navigate a swarm to trace and finally form certain moving target shapes, which demonstrate the assertion studied in Section 3.3. Additionally, we put a narrow channel between the swarm and the destination. When the swarm is passing through the channel, the shape controller devised in Section 4 can deform the shape to suit the channel and therefore take robots out of the channel.

### 5.1. Formation Convergence

**Figure 6:**
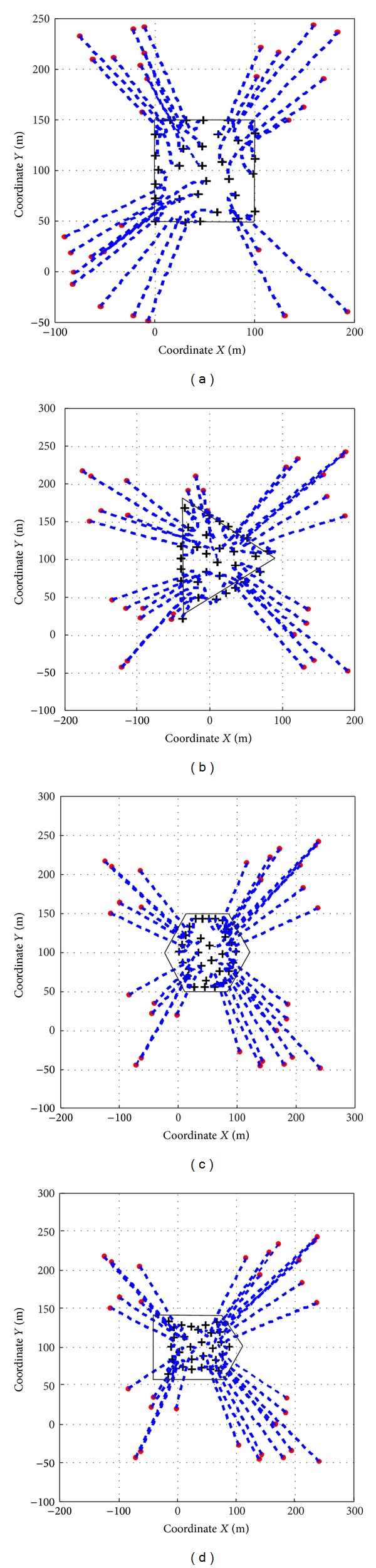
Formation convergence in different shapes. In the subfigures, red dots represent initial positions and black crosses represent final positions, while the thin lines denote the trajectories. The initial positions are uniformly distributed.

Figure 6 shows the motion of a swarm with 30 robots when they are trying to converge into four types of static shape. For this case, we select the parameters as magnitude factors *k*_*a*_ = 1, *k*_*r*_ = 2, *k*_*f*_ = 5, *k*_*m*_ = 15, *k*_*ac*_ = 1, and *k*_*o*_ = 10; maximal acceleration and velocity of the robot are 7 m/s^2^ and 4 m/s; reacting range control parameters *β*_*a*_ = 0.1, *β*_*ac*_ = 0.1, *β*_*r*_ = 0.1, *β*_*m*_ = 0.05 and *β*_*o*_ = 0.05.

Figures 6(a) and 6(b) use the basic attraction force definition as described in (8). It is clear that the robots are lingering back and forth near by the contour. This phenomenon illustrates that with the basic definition of *F*_*i*,*a*_ and relatively small shape area, some robots usually failed to step into the shape. This is because *F*_*i*,*a*_ decreases then the resultant repulsion force that generated from the robots in the contour repel them.

Figures 6(c) and 6(d) use the modified *F*_*i*,*a*_ defined in (45). The whole swarm can avoid chatter effect and converge into the predefined formation shape. The reason is that the modified *F*_*i*,*a*_ can keep the uniformity of the force distribution on both side of the boundary, so the attraction force will hold on till the robots get through into the shape; thereafter, *F*_*i*,*r*_ continuingly navigates them to stay within the shape.

### 5.2. Formation Transition

**Figure 7:**
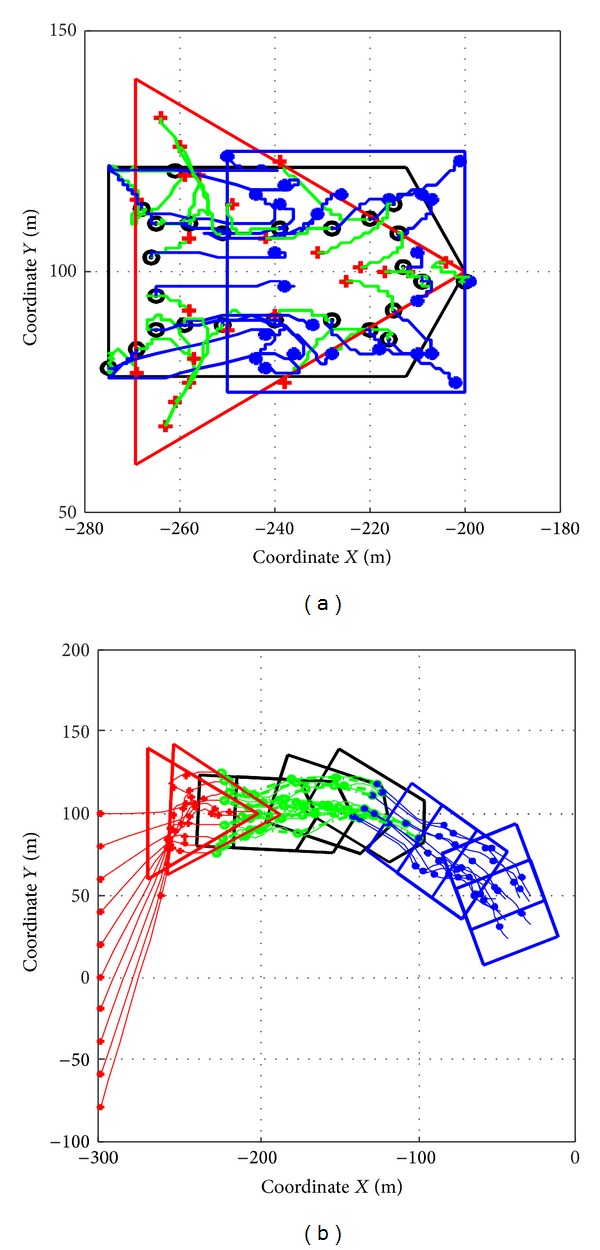
Simulation cases for shape transition in static and moving situations.

In real world implementations, formation transitions are very usual.  Figure 7 shows the simulation results about the behavior of a swarm with *N* = 10 robots. The swarm is subjected to a sudden formation transition. The parameters used here are the same as the ones used in Section 5.1.

We test formation transitions in both stationary and moving target shape scenarios. As is seen in Figures 7(a) and 7(b) the formation type transits in interval of 20 seconds from triangle via pentagon to square.

Figure 7(b) demonstrates that the control algorithm for stationary shape is also adaptable in moving formation control. By using theoretical assertions proposed in Section 3.3, the shape can follow arbitrary trajectory and the forces from the shape are calculated by (51) and (52), which is subjected to a relative reference framework moving with the shape.

### 5.3. Obstacle Avoidance

**Figure 8:**
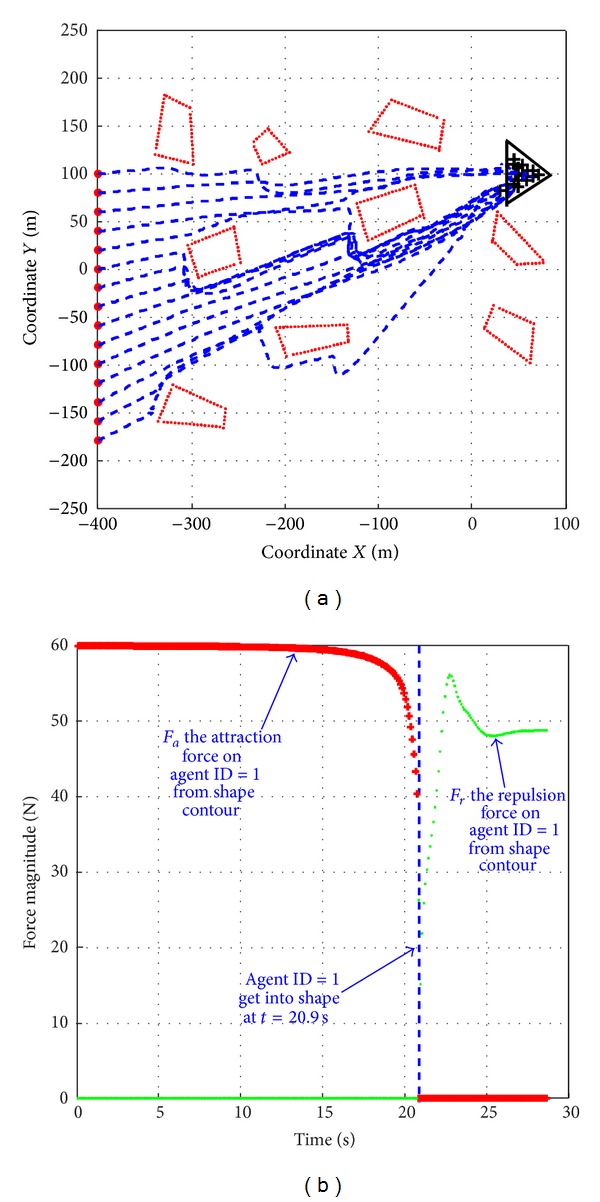
Obstacle avoidance in the complex terrain, using modified *F*_*i*,*o*_.

In this section, the velocity and actuator limitations are taken into account. As shown in Figure 8(a), there is a swarm of 15 robots in a line formation (represented as red dots) and the target shape (a triangle formation) is located far away from the line. There are several obstacles between the start line and the target shape. The blue dots denotes the paths of the robots and the black crosses are their final positions. The velocity and accelerate are limited to 4 m/s and 7 m/s^2^ respectively. *K*_*o*_ is set as 100, *β*_*o*_ = 0.01, and *R*_*s*_ = 10 m.

Figure 8(a) shows that all the robots that successfully escape avoid these obstacles and form the desired formation within 30s. By using the modified *F*_*i*,*o*_ defined in (39) together with (41), the robot getting closer to the obstacle boundary is dragged by a force parallel the boundary, so it can pass by the obstacles and avoid local minima during the whole process.

Figure 8(b) shows the change of *F*_*i*,*a*_ (the red curve) and *F*_*i*,*r*_ (the green curve) acting on robot ID = 1 when it gets through the formation contour boundary. We can figure out that with the modified *F*_*i*,*a*_ defined in (45), the force uniformity between *F*_*i*,*a*_ and *F*_*i*,*r*_ at time *t* = 20.9 s is ensured.

### 5.4. Shape Contour Controller

In this part the validity of the contour controller is demonstrated via the case of passing narrow channels. In order to make the swarm a damping system that can make the formation contour deform smoothly, the value of the parameters should be $\lambda_{c}=15,\;b_{c}=\sqrt{4\lambda_{c}+1}=7.8102,\;\zeta =\left(1/2\right)\sqrt{4+\left(1/\lambda_{c}\right)}=1.0083>1$, and $t_{s}=8/\sqrt{4\lambda_{c}+1}=1.0243\;s$.

**Figure 9:**
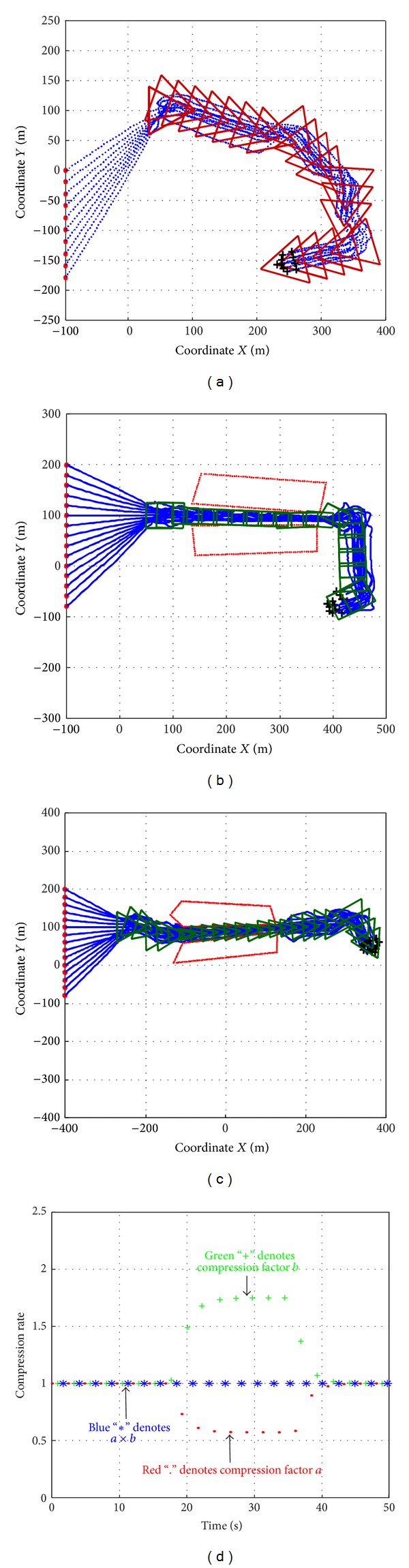
Simulation cases for moving target shape.

Figure 9(a) shows the motion of a swarm of 10 robots moving in a triangle formation. When there is no channel to get through, the controller keeps compression force *F*_*C*_ = 0 and therefore the shape contour is invariant.

In Figures 9(b) and 9(c), a swarm of 15 robots follows a square and a triangle formation respectively. When the swarm reaches the entrance of the channel, the formation, contour begins to suffer pressure *F*_*C*_ whose value is determined by (58), which deforms the contour into a compact one and navigates the whole swarm through the channel. After getting out of the channel, the tensile force *F*_*T*_ defined in (57) stretches the contour back to its original state.

Figure 9(d) shows the change of the compression ratios defined in (62). It is clear that the value of *a* × *b* (represented as blue stars) always equal 1, which means that the area of the formation shape is invariant. When the swarm is passing the channel, the behavior of the compression matrix which is described in (62) will follow the dynamic model defined in (60).

## 6. Conclusions

This paper presented a potential field-based approach for formation control of robotic swarms. We defined artificial forces in the form of exponential functions; therefore, the magnitude of the resultant force is constrained within reasonable range. The behavior of the swarm along with single robot was both analyzed. Realistic implementation considerations and adaptation of the control parameters have been investigated to enhance the robustness of the control algorithm. We additionally built a controller acting on the shape contour, making it autodeformable to prevent the member robots from collisions. Simulation results validate the efficiency of the proposed algorithm.

In this paper the potential field is generated by several wavefront expansion procedures starting at the contour of the formation shape or edges of obstacles. By following the flow of the negated gradient from the target shape, a trajectory is obtained to navigate each member of the swarm. Using such kind of resultant potential field is the fundamental reason that produces local minima near by the obstacle boundary [41]. To reduce the risk of such an impasse and to enlarge the maneuvering space of the robot, we highlight our future research on finding novel algorithms to generate potential field that is more flexible and less likely to produce local minima. One of the most promising trends is to make use of topological properties of the underlying environments. For instance, we are now working on the construction of sparse but adequate roadmaps such as Generalized Voronoi Diagrams (GVDs) and Equidistance Maps (EM). These spatial representation can be adopted to generate potential fields avoiding most local minima and providing reduced search space for navigation tasks.
